# Supplementary material for: Dual oxidase Duox and Toll-like receptor 3 TLR3 in the Toll pathway suppress zoonotic pathogens through regulating the intestinal bacterial community homeostasis in Hermetia illucens L
Source: PLoS One. 2020 Apr 30;15(4):e0225873. doi: 10.1371/journal.pone.0225873 (PMC7192390; doi:10.1371/journal.pone.0225873)
Supplement: S1 Fig — (PDF) [file pone.0225873.s001.pdf]

Figure S1

ATGGATGTTCCTTGTTGGAAGATAGTTCTCTTGCTCGTCGGAGTCATAAGATTTACAGAA
M D V P C W K I V L L L V G V I R F T E
CCGACCGACGTTCTACCAAATGAAACTGGCTTTAGTCGAATAGAAAAGCAACGATACGAT
P T D V L P N E T G F S R I E K Q R Y D
GGCTGGTACAACAATTTGGCTCATCCAGATTGGGGATCTGTTGACAGTCACCTGGTCCGA
G W Y N N L A H P D W G S V D S H L V R
AAGGCACCCCCTGCATATTCCGATGGGGTTTATGCCTTAGCAGGTATCAACCGACCATCA
K A P P A Y S D G V Y A L A G I N R P S
CCGCGACGATTGATGCGTCTTTTTATGCGGGGTCAAGATGGCTTAGGCTCCAAAACAAAT
P R R L S R L F M R G Q D G L G S K T N
AGGACCGCCCTACTTGCATTCTTCGGGCAGGTGGTAGCCAACGAAATCGTTATGGCTTCC
R T A L L A F F Q G V V A N E I V M A S
GAATCTGGTTGTCCCATAGAAATGCACTGAATTGACATTGAAAAATGCGATGATATGTAC
E S G C P I E M H R I D I E K C D D M Y
GACAAAGAATGTCGAGGAAATCGTTATATTCCATTCTTGAGAGCGGCATATGATCGAGAT
D K E C R G N R Y I P F L R A A Y D R D
ACTGGACAGAGCCCAAATGCTCCAAGAGAACAGGTGAACCAAATGACTGCTTGGATTGAT
T G Q S P N A P R E Q E N Q M T A W I D
GGCAGTTTCATCTATAGTACGTCGGAGGCATGGCTGAATGCTATGCGATCATTCCAGAAT
G S F I Y S T S E A W L N A M R S F Q N
GGTTCGTTGCTGACCGAGAAGGAAGGTCGCATGCCAATCAGGAACACAATGCGAGTTCCA

G S L L T E K E G R M P I R N T M R V P
CTTTTCAATCATCCACTACCGCATGTGATGAGGACCTTAAGTCCGGAGCGATTATTCTTA
L F N H P L P H V M R T L S P E R L F L
CTCGGTGACCCGCGAACTAATCAAAATCCTGCTCTTCTCTCATTCGGGATCCTGTTCCTC
L G D P R T N Q N P A L L S F G I L F L
CGATGGCATAACACTTTGGCGAAACGTATTAAACGCCAACATCCCGACTGGAGTGACGAA
R W H N T L A K R I K R Q H P D W S D E
GACATATTCCAGCGAATCCGTCGCATTGTTATAGCTAGTCTTCAAAATATAATTTTTTAT
D I F Q R T R R I V I A S L Q N I I F N
GAATATTTGCCGGAATTTTTGGGCTCTAAAATGCCTGAATATACGGGGTACAACCAAGAT
E Y L P E F L G S K M P E Y T G Y N Q D
GTCCATCCTGGCGTTGGTCATCTTTTCCAAGCGGCAGCTTTCCGATTTGGTCATACCATG
V H P G V G H L F Q A A A F R F G H T M
ATACCACCAGGAATTTACAGACGTGACGCTAAGTGCAACTTTAGGAAAACGTCCATGGGG
I P P G I Y R R D A K C N F R K T S M G
TATCCGGCTATGCGTCTGTGTTCGACTTGGTGGGATTCGAATGACTTTCTGGCCAAGTCA
Y P A M R L C S T W W D S N D F L A K S
TCAGTCGAAGAGATCCTTATGGGTCTTTCATCGCAAATTTcCGAGCGAGAGGATCCGGTA
S V E E I L M G L S S Q I S E R E D P V
CTATGCTCCGATGTGCGCGATAAGCTGTTCGGTCCGATGGAGTTCACAAGAAGAGATTTG
L C S D V R D K L F G P M E F T R R D L
GGAGCTTTGAACATTATGCGAGGAAGAGATAATGGACTCCCTGATTATAACACAGCACGT

G A L N I M R G R D N G L P D Y N T A R
GAAGCCCTAGGACTATC AAAAAGGAAAACCTGGGAAGAGATCAATCC AGAACTATTTAAG
E A L G L S K R K T W E E I N P E L F K
GCTAAGCCGGAATTATT AAAAGCTCTAATTTCCGTTTACAACAACCA ATTGGACAACATT
A K P E L L K A L I S V Y N N Q L D N I
GACGTCTACGTAGGCGG TATGCTGGAATCCTACGGAGGTCCTGGCGA ACTATTCACGGCT
D V Y V G G M L E S Y G G P G E L F T A
GTCATCAAGGAACAATT CATTCGAATCCGAGATGCTGACCGATTCTGG TTCGAGAATAAT
V I K E Q F I R I R D A D R F W F E N N
CGCAGCAATATGTTCAC CGCTGAAGAAATCGCAGAACTTCGCAAGAT CACCCTTTGGGAC
R S N N F T A E E I A E L R K I T L W D
GTGACCGTCAACAGCAC CGACATAAAACCAGGTGACATCCAGAAGAA CGTATTCATGTGG
V I V N S T D I K P G D I Q K N V F M W
CATAAAAATGATCCCTG CCCGCAACCAATGCAGCTAAACGCTTCTGA GTTAGAACCATGC
H K N D P C P Q P M Q L N A S E L E P C
AGCTACTTGCAAGGATA TGATTATTTCTCAGGATCAGAGCTGACTTT CATTTATGCCTGT
S Y L Q G Y D Y F S G S E L T F I Y A C
GTCTTCCTTGGTTTTGT TCCGATATTGCTCGCTGGAGCAGGATATTGT GTTGTAAAGTTA
V F L G F V P I L L A G A G Y C V V K L
CAAAACAGCAAACGAAG GAAACTGAAGATACGCCAGGAAGCAATTAA GTCCAGTGCTCAA
Q N S K R R K L K I R Q E A I K S S A Q

CATAAAGGATCTGTGGATAAGAT GATAGCGCGGGAATGGTTGCATGCTAATCACAAGCGA
H K G S V D K M I A R E W L H A N H K R
TTGGTTACTGTGAAATTTGGTCC TGAAGCTGCAATATACACAGTCGACCGTAAAGGCGAG
L V T V K F G P E A A I Y T V D R K G E
AAATTAAGGACATTTTGCTTGAA AAATGTCGATTTGGTCACCGTGGAAGAATCTCAAATA
K L R T F C L K N V D L V T V E E S Q I
ACAAATTTAAGGAAGAAACCATA TCTCCTGATAAGAATTCATAACGATCATGATTTGGTG
T N L R K K P Y L L I R I H N D H D L V
CTTGAATTAGATTCAGTGAATTC TCGAAGGAAGTTTATCAAGAAATTAGAAGATTTCCTG
L E L D S V N S R R K F I K K L E D F L
CTGCTACATAAAAGAGAGTTGAC TGTAATTGAGGTTCCTAAAGACATCATGCTGACTCGT
L L H K R E L T V I E V P K D I M L T R
GCGGAAACTCGAGAAAGAAGACA AAAGCGTCTGGAATACTTCTTCAGAGAAGCATATGCC
A E T R E R R Q K R L E Y F F R E A Y A
CTTACCTTCGGCCTAAAACCTGG CGAGCGCAGACGCCGTTCAGACACCTCAGCCGATGGC
L T F G L K P G E R R R R S D T S A D G
GAAGTCATGACCGTCATGCGAAC TAGCCTTTCCACTGCCGAATTCGCCGCAGCGCTCGGG
E V M T V M R T S L S T A E F A A A L G
ATGAAATCAAACGACATGTTTGT TCGAAAAATGTTCAATATTGTTGACAAGGATCAAGAC
M K S N D M F V R K M F N I V D K D Q D
GGACGAATATCCTTCCAAGAGTT CCTGGAGACTGTAGTACTATTTTCACGAGGGAAGACC
G R I S F Q E F L E T V V L F S R G K T
GAGGATAAGTTGCGAATCATTTT TGATATGTGTGATAATGATCGGAATGGAGTAATAGAC

E D K L R I I F D N C D N D R N G V I D

AAGGAAGAACTGAGCGAAATGAT GCGATCCTTGGTAGAGATTGCAAGAACCACTAGTCTT

K E E L S E M M R S L V E I A R T T S L

AGGGATGACCAGGTTACCGAGCT CATTGACGGAATGTTCCAGGATGTAGGCTTGGAGCAT

R D D Q V T E L I D G N F Q D V G L E H

AAGAACCATTTGACTTACCAAGA CTTTAAGCTTATGATGAAGGAGTATAAGGGAGACTTT

K N H L T Y Q D F K L M M K E Y K G D F

GTCGCGATCGGGCTGGATTGCAA AGGAGCGAAACAAAATTTCTTGGATACATCAACGAAT

V A I G L D C K G A K Q N F L D T S T N

ATCGCACGAATGACGTCGTTTCA TATTGAACCTGTGCAGGATATTCATCGACATTGGCTG

I A R N T S F H I E P V Q D I H R H W L

CAGAAGCAATGGGATAGTTACAC AACTTTCTTGGAAGAGAATAGACAAAATATCTTTTAT

Q K Q W D S Y T T F L E E N R Q N I F Y

TTATTTTTATTTTATGTGATCAC GATCGTACTATTTGTTGAGAGGTTCATACATTACTCC

L F L F Y V I T I V L F V E R F I H Y S

TTTATGGCTGAGCATACTGATCT GAGGCATATTATGGGAGTGGGCATTGCGATAACTCGT

F M A E H T D L R H I M G V G I A I T R

GGATCGGCTGCATCACTATCATT TTGCTACTCTCTGTTACTGCTAACAATGTCGAGAAAT

G S A A S L S F C Y S L L L L T M S R N

CTAATAACAAAACTGAAAGAGTT CCCGATCCAACAATACATCCCCTTGGACTCTCATATC

L I T K L K E F P I Q Q Y I P L D S H I

CAGTTCCATAAAATCGCCGCCTG TACAGCCCTGTTTTTCTCGCTCCTCCATACAGTCGGT

Q F H K I A A C T A F L F S L L H T V G

CACATAGTGAATTTCTATCATGT TTCGACCCAATCCGTTGAAAATCTTCACTGCCTCACA

H I V N F Y H V S T Q S V E N L H C L T

AGAGAAGTTCACTTCGCGTCCGA CTATAAGCCTGATCTGACCTTCTGGTTGTTCCAAACA

R E V H F A S D Y K P D L T F W L F Q T

GTTACTGGTCTAACCGGTGTCCT TCTCTTCATCATAATGGCAGCGATCTTCGTTTTCGCC

V T G L T G V L L F I I M A A I F V F A

CATCCGACCATCAGGAAGAAGGC ATACAAATTCTTCTGGAACGTTCATGCTCTATATATC

H P T I R K K A Y K F F W N V H A L Y I

CTGCTTTATATTTTGTGTTTGAT TCATGGGCTGGCGAGACTTACAGGACCACCGAGATTC

L L Y I L C L I H G L A R L T G P P R F

TGGATGTTCTTCATTGGACCGGG AATTATTTACACCTTGGATAAGATCGTCTCACTACGT

W M F F I G P G I I Y T L D K I V S L R

ACAAAATACATGGCTCTAGACGT CATGGAAACCGATCTTCTCCCATCCGACGTGATCAAA

T K Y M A L D V M E T D L L P S D V I K

AT CAAGTTCTACCGTCCGCCCAATCTAAAATACCTCTCCGGACAATGGGTTCGTCTATCC

I K F Y R P P N L K Y L S G Q W V R L S

TG CACGGCCTTGCGACCAACCGAAATGCATAGCTTTACTCTTACATCTGCTCCACATGAA

C T A L R P T E N H S F T L T S A P H E

AA CTTCCTAAGCTGCCATATCAAGGCGCAAGGACCATGGACATGGAAGCTTCGGAACTAT

N F L S C H I K A Q G P W T W K L R N Y

TT CGATCCGTGCAACTACAACCCGGAGGATCAACCGAAAATCCGTATCGAAGGTCCCTTT

F D P C N Y N P E D Q P K I R I E G P F

GG TCTCACTCCATATGCATCGATTCTCAACGATTTGGTATTTGGAACTAGCACGAATCGG

G V T P Y A S I L N D L V F G T S T N R

TA CTCAGGAGTGGCTTGCAAGAAGGTCTACTTCTTGTGGATTTGTCCGTCGCATAAGCAT

Y S G V A C K K V Y F L W I C P S H K H

TT CGAATGGTTCATCGATGTTTTGCGCGATGTTGAGAAGAAGGATGTTACCAATGTATTA

F E W F I D V L R D V E K K D V T N V L

GA AATTCATATTTTCATAACACAATTTTTCCATAAATTCGATTTAAGGACTACAATGCTG

E I H I F I T Q F F H K F D L R T T M L

TA TATTTGTGAAAACCACTTCCAGAGGTTGGCTAAAACTTCAATATTTACGGGACTTAAG

Y I C E N H F Q R L A K T S I F T G L K

GC TGTTAACCATTTTGGACGCCCTGATATGTCGAGCTTCCTCAAGTTTGTTCAGAAGAAA

A V N H F G R P D N S S F L K F V Q K K

CA TTCATATGTCTCAAAAATTGGTGTATTCTCCTGTGGACCGCGCCCCTTAACCAAGAGT

H S Y V S K I G V F S C G P R P L T K S

GT AATGTCGGCATGTGATGAAGTGAACAAAGGACGCAAGTTACCATATTTCATTCACCAT

V M S A C D E V N K G R K R P Y F I H H

TT CGAGAACTTTGGTTAG

F E N F G
