# Supplementary figures and images for: Dual oxidase Duox and Toll-like receptor 3 TLR3 in the Toll pathway suppress zoonotic pathogens through regulating the intestinal bacterial community homeostasis in Hermetia illucens L
Source: PLoS One. 2020 Apr 30;15(4):e0225873. doi: 10.1371/journal.pone.0225873 (PMC7192390; doi:10.1371/journal.pone.0225873)

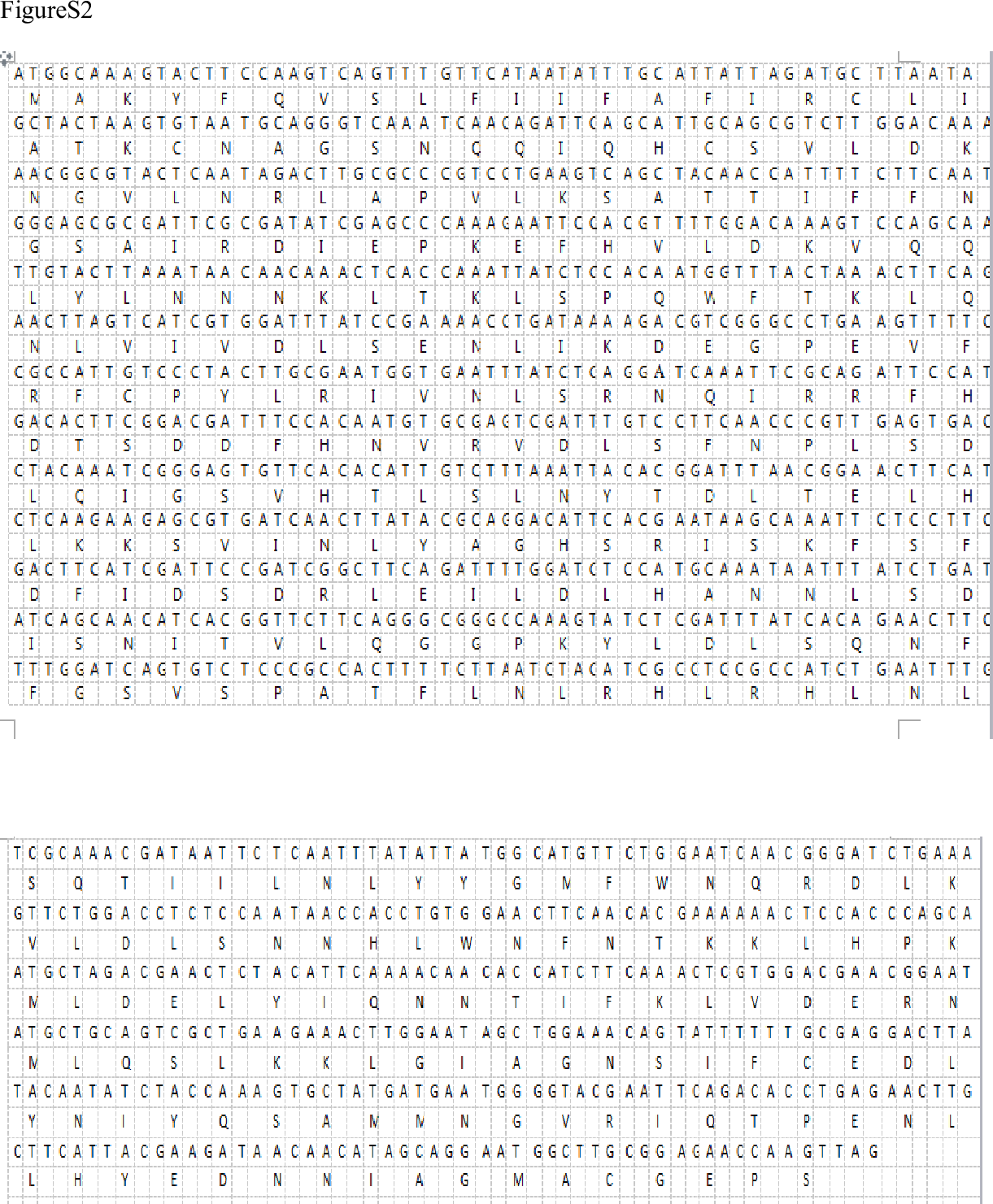

Supplement: S2 Fig — (TIF) [file pone.0225873.s002.tif]

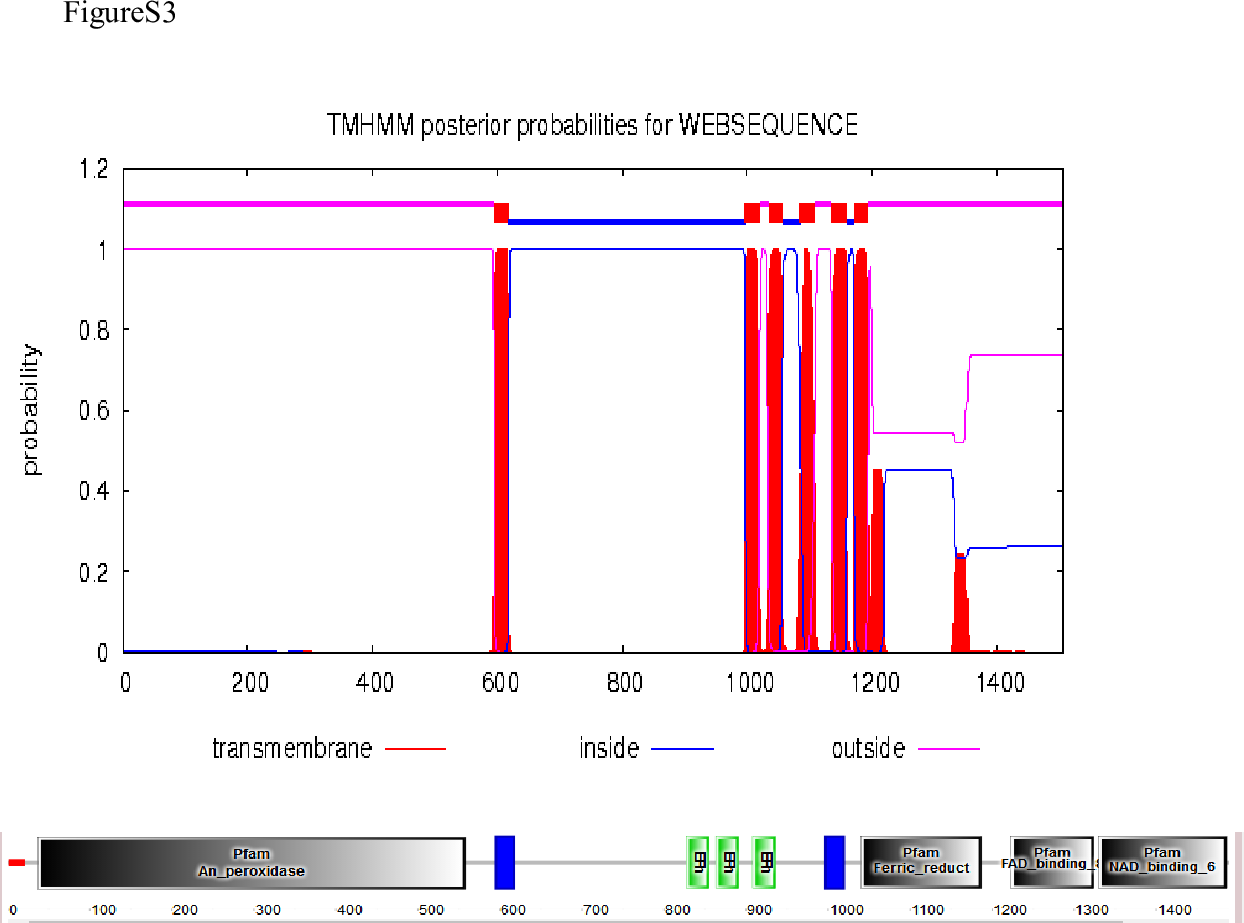

Supplement: S3 Fig — (TIF) [file pone.0225873.s003.tif]

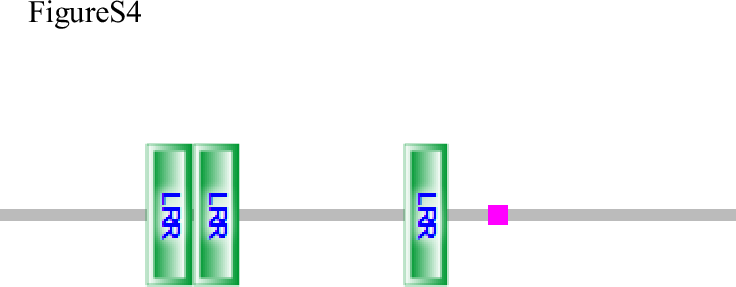

Supplement: S4 Fig — (TIF) [file pone.0225873.s004.tif]

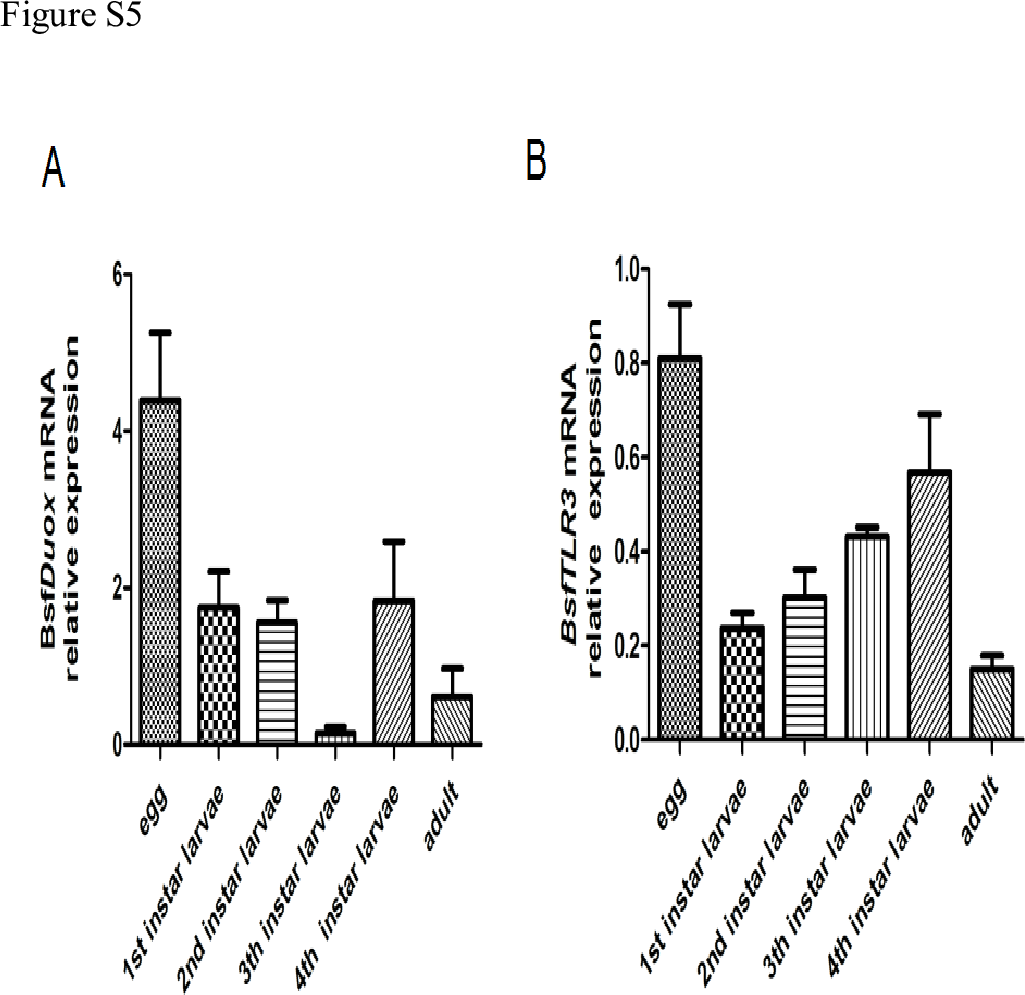

Supplement: S5 Fig — (TIF) [file pone.0225873.s005.tif]

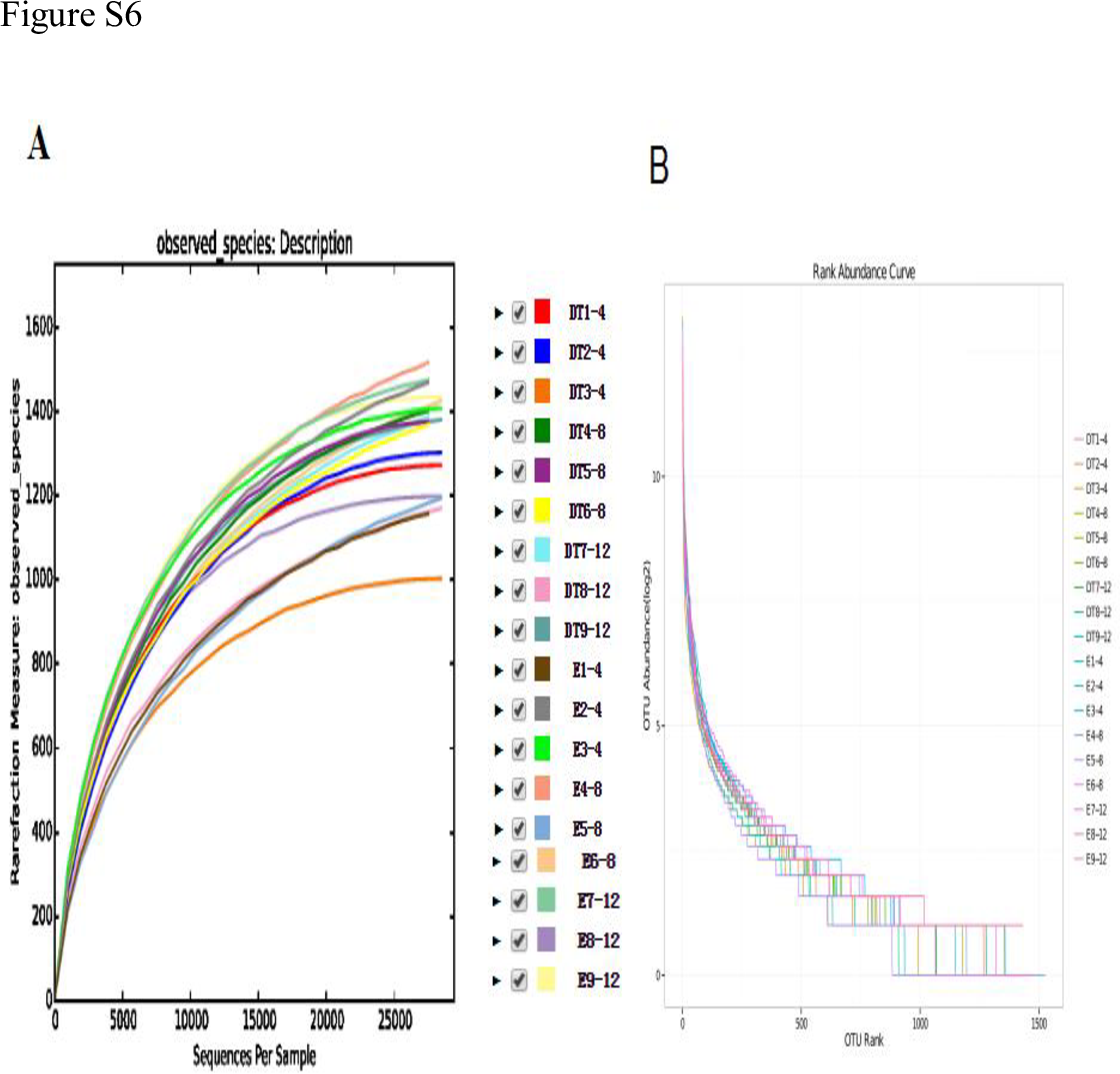

Supplement: S6 Fig — (A) Rarefaction curve. (B) Rank-abundance curves. (TIF) [file pone.0225873.s006.tif]

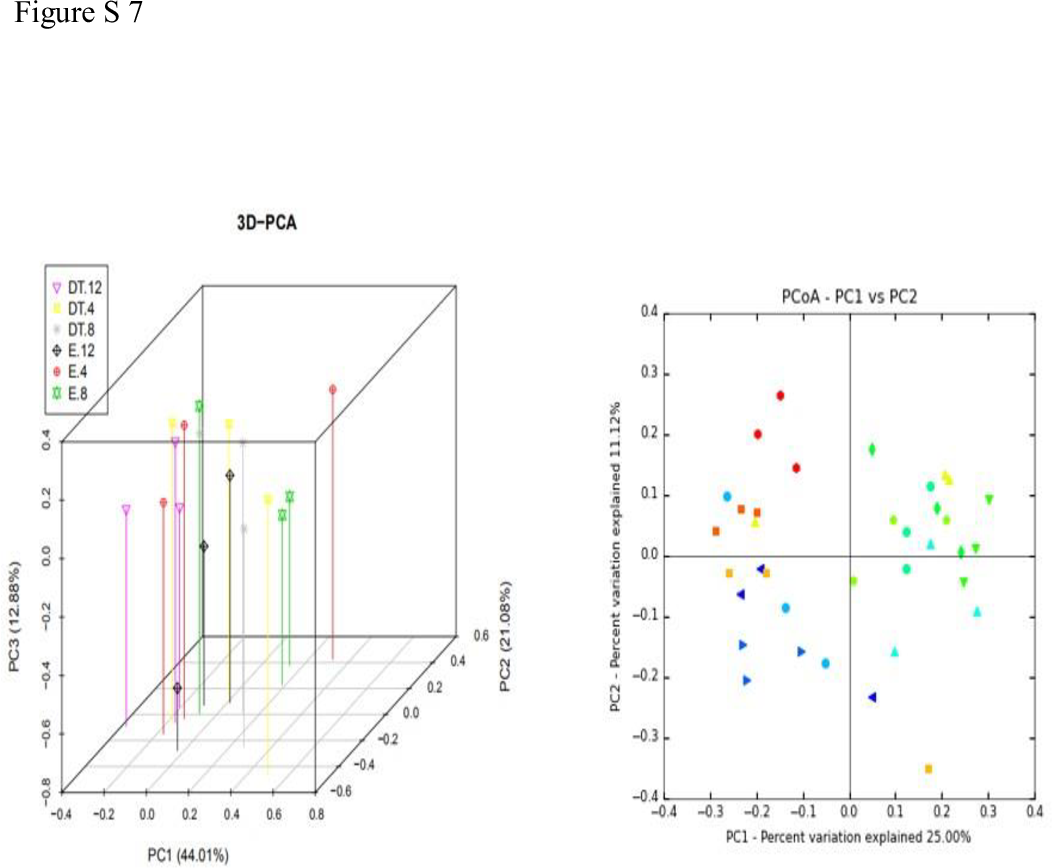

Supplement: S7 Fig — The result showed a separation of ds-Duox-TLR3 and ds-egfp-treated samples along the first two axes, which explained 44.01% and 21.08% of the data variation, respectively. (A) 3Dscore plot of the ds-Duox-TLR3 RNAi and ds-egfp RNAi sample (B) 2D biplots on PC1–PC2 plane overlapping scores and loadings. (TIF) [file pone.0225873.s007.tif]
