# Supplementary material for: Dual oxidase Duox and Toll-like receptor 3 TLR3 in the Toll pathway suppress zoonotic pathogens through regulating the intestinal bacterial community homeostasis in Hermetia illucens L
Source: PLoS One. 2020 Apr 30;15(4):e0225873. doi: 10.1371/journal.pone.0225873 (PMC7192390; doi:10.1371/journal.pone.0225873)
Supplement: S1 Table — (DOCX) [file pone.0225873.s008.docx]

Table S1 Primers used in this study

| Application | Gene name | Primer name | Primer sequence(5'-3') |
| --- | --- | --- | --- |
| qPCR  analysis | *BsfDuox* | *Duox*F | CAGACGCCGTTCAGACAC |
|  |  | *Duox*R | ATCGCATCATTTCGCTCA |
|  | *BsfTLR3* | *TLR3*F | CGCCATTGTCCCTACTTG |
|  |  | *TLR3*R | AACCGTGATGTTGCTGAT |
|  | *BsfCecropin* | *Cecropin*F | AAGAGTTCGTGATGCTGGTA |
|  |  | *Cecropin*R | ATTGGCTCCTTGTTGTGC |
|  | *BsfDLP4* | *DLP4*F | ACCTGCAAGGATTCTGGG |
|  |  | *DLP4*R | GCTGTGCCGCTAAACTCG |
|  | *BsfDefencin* | *defencin*F | ACGACTCCACATCTGGCG |
|  |  | *defencin*R | GTCATCGCACCATCCTCC |
|  | *BsfUbiqiutin* | *Ubiqiutin*F | AGCATAGAAATGACGGGA |
|  |  | *Ubiqiutin*R | AATTGGTGGATCGACAGC |
|  | *BsfStomoxynZH1* | *StomoxynZH1*F | AACTCGCCATTGTCCTTT |
|  |  | *StomoxynZH1*R | CTCCAGCTAATCCTTCCAC |
|  | *Bsfdorsal* | *dorsa*lF | ATGAATGCGAAGGACGAT |
|  |  | *dorsal*R | CCTCACGAGCACGAAGTG |
|  | *Bsfdif* | *dif*F | ATGAATGCGAAGGACGAT |
|  |  | *dif*R | CCTCACGAGCACGAAGTG |
|  | *Bsf*b*-actin* | *b-actin*F | AAACCTTCAACGCCCCAGC |
|  |  | *b-actin*R | GGCGTGTGGAAGAGCATAACC |
|  | 16SDNA  gene-specific  primers | 16SDNAgF | ACTCCTACGGGAGGCAGCAG |
|  |  | 16SDNAgR | ATTACCGCGGCTGCTGG |
|  | *b-actin* DNA  primers | *b-actin*  DNAF | AGGCTCCACTCAACCCAA |
|  |  | *b-actin*  DNAR | GCCAAATCCAGACGCAAG |
| dsRNA  synthesis | *dsBsfDuox* | *dsBsfDuox*F | TAATACGACTCACTATAGGGGGGTTCGTCTATCCTGC |
|  |  | *dsBsfDuox*R | TAATACGACTCACTATAGGGCCTGAGTACCGATT |
|  | *dsBsfTLR3* | *dsBsfTLR3*F | TAATACGACTCACTATAGGGCGCCATTGTCCCTACTTG |
|  |  | *dsBsfTLR3*R | TAATACGACTCACTATAGGGAACCGTGATGTTGCTGAT |
|  | *dsegfp* | *ds-egfp*F | TAATACGACTCACTATAGGGTCGTGACCACCCTGACC |
|  |  | *ds-egfp*R | TAATACGACTCACTATAGGGTCACCTTGA |
