# Supplementary material for: Dual oxidase Duox and Toll-like receptor 3 TLR3 in the Toll pathway suppress zoonotic pathogens through regulating the intestinal bacterial community homeostasis in Hermetia illucens L
Source: PLoS One. 2020 Apr 30;15(4):e0225873. doi: 10.1371/journal.pone.0225873 (PMC7192390; doi:10.1371/journal.pone.0225873)
Supplement: Attachment — Submitted filename: Response to reviewers.docx [file pone.0225873.s009.docx]

Dear Editor,

Journal of PLoS One

**Subject: Response to Reviewers’ Comments**

## Kindly refer to your email sent on 2019/12/30 regarding the reviewers’ comments on our manuscript ID WM-19-31548 entitled "Dual oxidase Duox and Toll-like receptor 3 TLR3 in the Toll pathway suppress zoonotic pathogens through regulating the intestinal bacterial community homeostasis in *Hermetia illucens* L.". Based on his/her kind comments, we have carefully modified the manuscript. We submit the manuscript with track and no track version according to you requirements. The sequencing data of black soldier fly larvae gut bacteria 16S rDNA high-throughput sequencing has been uploaded. Serial number of original sequence data: bioprojectID PRJNA600829 and SRP247530 were added in Materials and Methods section of text. We have thoroughly revised as per suggestions of reviewers. The English language has been revised by a native English speaker scientist Professor Jeffery K. Tomberlin from Texas A &M University, USA .Thank you very much for the encouragement and providing very useful suggestions to improve the quality of our manuscript. We hope that the revised-manuscript now will meet the standards for publication in your esteemed journal.

Thank you very much.

Sincerely yours,

**Corresponding author**

Dr. Zhang Jibin

Professor

College of Life Sciences and Technology,

Huazhong Agricultural University,

Wuhan 430070, P.R. China

E-mail: [zhangjb@mail.hzau.edu.cn](mailto:zhangjb@mail.hzau.edu.cn)

Reviewer #1: This study focused on the mechanism black soldier fly larvae used to inhibit two pathogens, S. aureus and Salmonella. To do so, two immune genes were silenced, upon which two symbionts were observed to decrease in abundance and inhibiting effects on the pathogens were reduced. This demonstrated that the immune genes under study play a role in the pathogen reducing effect of native BSFL.
The paper brings important and new information and is highly relevant for the application of BSFL as waste converter, since waste streams can contain the pathogens under study. However, the language use was very poor and disappointing, and it reduced the readability of the paper. The many language errors deduct the reader from the content and devaluate the manuscript. As a reviewer, I corrected a number of language errors, but certainly not all of them. I strongly recommend professional language editing. On some places in the paper, statements were formulated too general and conclusions were drawn too strong in my opinion, i.e. based on evidence that was not substantial enough to make that conclusion. Also some elements are missing in the materials and methods section. I believe this manuscript can become a very meaningful paper as it contains interesting results but it needs major revisions.

| **Reviewer Comment**  Reviewer #1 | **Author' Response** | **Revised Text** |
| --- | --- | --- |
| L17-19: are confusing and appear to say that much more is legally allowed in Europe than is currently the case. Please rephrase. | Thank you for your suggestions. I have repharased it . | L17-19 |
| L 21: BSF larvae inhibit these zoonotic pathogens”. Here to, the claim is too general | Thanks for your suggestion, we have revised it . | L21-22 |
| L28: “Providencia and” and“Dysgonomonas intestinal”: spaces are missing | Thanks for your suggestion, we revised it. | L29 |
| L 40-41:“whose larvae consume various organic wastes of environmental concern”. This is a strange formulation | Thanks for your suggestion. We have revised it. | L40-41 |
| L55:Replace“phenoloxidase” by “phenol oxidase” | Thanks, we have revised it | L52 |
| L57:“GNBP3 and PGRP-SA”: please clarify abbreviation. | Thanks, we have revised it | L57 |
| L62: the explanation of a Toll-like receptor as written here may not be clear to all readers.Please try to be more clear | Thanks for your suggestion. we have revised it. | L62-69 |
| L66: “With routine microbial burdens, such as those found in the absence of infection, the Toll pathway at low activation levels.” A verb is missing in this sentence. | Thanks for your suggestion. We have revised it . | L67 |
| L75：Replace “Microbial flora modulate” by “The microbiota modulates”. | Thanks for your suggestion. We have revised it . | L75 |
| L78-79：ROS“to protect themselves against pathogenic microorganisms, such as bacteria and fungi”. And what about entomopathogenic viruses?: | Thanks for your suggestion, we have revised it. | L81 |
| L79: “Kumar et al. (2010) [11] and Mitochondria something is wrong with the construction of this sentence. | Thanks, we have revised it in text. | L83-85 |
| L 88：Please include the following references here: https://doi.org/10.1128/AEM.01864-18,  https://doi.org/10.1007/s00248-018-1286-x. | Thanks, we have revised it in text. | L94 |
| L102: replace “whether BSFL reduces suppression on zoonotic pathogens after BsfDuox-TLR3 RNA interference 104 (RNAi)” by whether suppression of zoonotic pathogens by BSFL is reduced after …”. | Thanks, I have rewritten it in text. | L111 |
| L110:Please include how you sterilized the feed | Thanks for your suggestion, we have revised it in text.it was done by autoclavation, I am sure that nutrients was not breakdown and the larvae were not affected by the sterilization process beacuase that's what we used to do in the lab, and it didn't affect the growth of the black soldier flies | L120 |
| L111: what type of bran was use here? | Thanks for your suggestion, we used wheat bran to feed the black soldier flies.we have revised it in text. | L120 |
| L112: Please indicate the duration of each washing step so that other researchers can repeat the work | Thanks, we have revised it in text. | L122-L123 |
| L 115: How was homogenisation performed? Equipment? Process parameters? | Thanks, we have revised it in text. | L126 |
| L 120: “equal amounts of sterile distilled water”: equal to what? How did you determine this? Please specify. | Thanks, we have revised it in text. | L131 |
| L124:please mention supplier of the medium | Thanks for your suggestion, we have added supplier in text. | L135-L136 |
| L138: Did you perform the appropriate confirmation tests on the presumptive colonies as is to be done for Baird-Parker medium?In other words, how specific was your counting method? | The experiment used a selective medium, in which only the *Staphylococcus aureus* could grow black colonies, and other bacteria could not grow on a selective plate. |  |
| L 130 to 136 and 145 to 150 are the same. | Thanks for your suggestion, we have revised it in text. | L154-158 |
| L 154: same remark as for Staphylococcus: did you perform confirmation tests, as is a standard rule in microbiology? | The *Staphylococcus aureus* and *Salmonella* colonies are counted according to the method in National standards for food safety.it is a standard rule in microbiology in China. National standards GB4789.10–2010 for *Staphylococcus aureus* detection*.* National standards GB4789.4–2016 for *Salmonella* spp detection. |  |
| L 139 and 155: you mention results were expressed in cfu/g but in the figure you mention log cfu/g. Therefore, mention log cfu/g in your text as well. | Thanks, we have revised it in text. | L151,L158 |
| L167: “data not published”Why not refer to https://doi.org/10.1038/s41422-019-0252-6 | Thanks for your suggestion, we have revised it in text. | L174 |
| L 178: “Third-instar larvae (8 days old) were fed with an artificial diet containing concentrated microbe solution (1×10^8^ CFU/ml),”: You mention the cell density of the inoculation suspension, but you do not mention how much of this solution was used | Thanks for your suggestion. Per third-instar larvae (8 days old) were fed with 1 ml solution containing concentrated microbe (1×10^8^ CFU/ml). We have revised it in text. | L194-195 |
| L184:“The resulting bacterial counts in each sample were adjusted to 1×10^8^ CFU/ml by aseptic distilled water.” As I understand, this means that you prepared the cell suspension, you plated and incubated it and then counted the colonies. How long did this step take? If it took a few days (for incubation), how can you be sure that in the suspension all cells remained alive? Typically in microbiology, to assess the inoculum size, turbidity is measured and a | We did a preliminary test at first. For the first sample solution, the bacterial clones were calculated by counting plate.It take several days and turbidimetric OD value of the preliminary sample is measure by photometer. For formal sample measures the turbidimetric OD value to determine the number of bacteria refering to the preliminary results. | L188-L194 |
| L185: The larvae fed with a 5% sucrose diet only served as the control.” Why did you use a sucrose as control? Can you exclude that the sucrose did not affect the growth and the intestinal microbiota of the larvae and hence that this was a good control? Please explain. | 5% surose water has a little sugar, which can provide nutrition for the black soldier fly, but does not affect the composition of the intestinal microflora, which is demonstrated in *Drosophila*. |  |
| L188:“The microorganisms used in this study were pathogens S. aureus and Salmonella spp.” This sentence is redundant because you mentioned this many times earlier in the text. Please delete the sentence. | Thanks, we have revised it in text. | L198 |
| L 191: Is a sample of three larvae representative for the whole batch? Please prove so. | We chose all the same sized black soldier fly, three of which are representative for the whole batch.The guts of three black soldier flies are just enough to extracted the RNA which is for later experiments. | L191 |
| L195: Language problem. Replace “The amplification program was consist of” by The amplification program consisted of” | Thanks, we revised it in the text. | L205 |
| L 208: What do you mean with “rapidly”? How long did the dissection of one larvae take maximally? | We disect the larvae take five to ten seconds, so we used the rapidly in the sentense. | L221 |
| L209 “The dissected intestines were ground with PBS.” Explain how. Can you assure that no ROS were lost during grinding? | The dissected intestines were ground with PBS solution in（0.01 M，1ml） in the grinder.Losted ros are negligible | L222 |
| L 221:You mention that you measure the tissue and centrifuged it. Do you mean the ground tissue? If so, please add the word “ground”. | Thanks for your suggestion, we have revised it in text. | L227 |
| L207: says you measured TOTAL in vivo ROS, but in Line 209 you say you took the dissected intestines. Can you be sure that ROS only occur in the intestines of the larvae and thus that by taking the intestines you indeed determine all ROS? Can there be ROS in other tissues of the larvae as well and would the title in Line 207 better be “Measurement of intestinal ROS”? | Thanks for your suggestion,The major tissues that produce ROS in the black soldier fly was gut,other tissue produce very little ROS. we have revised it in text. | L220 |
| L224-225: “primer pairs used in dsRNA synthesis are shown in Supplemental Table 1.1 μg PCR product was used”. Some words appear to be missing here: is 1.1 the number of the Table you refer to or is it the amount of PCR product used? | “primer pairs used in dsRNA synthesis are shown in Supplemental Table 1. 1 μg PCR product was used. These were two sentense . There's a punctuation in the middle. | L238-L239 |
| L 281: “Each experiment was repeated three times”. Formulated this way, a reader may think that each experiment was performed a first time, and then later, AFTER the first experiment a second time, and again later a third time and finally, after the third time a fourth time. This is was is meant with REPEATED three times. However, this is not consistent with what you write in Line 161, where you say that the experiment was PERFORMED three times. Please make sure this is consistent in your text. | Thanks for your suggestion, we have revised it in text. | L296 |
| L300:“using the sterilized feed”: Why did you do this experiment in sterile feed? Please explain this choice in the paper. Did you expect that the attenuation would not work when the feed would not be sterilized? What is the possible impact of the absence of the background microbiota in the feed on the results? | Compared with pig manure,using the sterilized feeds with *Staphylococcus aureus* and *Salmonella* could eliminate the interference of other bacteria.I predicted that background microbiota in the feed could inhibits *S. aureus* and *Salmonella spp and* reduce the impact of black soldier fly on *Staphylococcus aureus* and *Salmonella* spp *.* | L318-L320 |
| L304：Replace “BSFL reduced the inhibition of *S. aureus* at all time points, and the interference effect was maintained” by “the inhibition by the larvae of:*S. aureus* was reduced at all time points and the effect was maintained”, if this is what you mean. | Thanks for your suggestion, we have revised it in text. | L325-L326 |
| L336:Replace “Infection of zoonotic pathogens” (which means that the pathogens get infected) by “Infection of zoonotic pathogens” (which means that the pathogens are infecting other organisms, here the larvae, I guess this is what you mean). | Thanks for your suggestion, we have revised it in text. | L362 |
| L366: “beneficial symbiont bacteria”: your data are based on the statement that Providencia and Dysgonomonas are symbionts. I have two questions here: (1) provide ample references from literature that these genera are indeed symbionts for BSFL to prove your statement and (2) can you assure you did not miss other symbionts? What if there are other symbionts in BSFL that are not discovered or described yet and that you do not take into account and that react completely different than the two you mention in your study? | Thanks for your suggestion, we have provide two references in text.  There are other symbionts in the black soldier flies,according to the results of sequencing, only these two types of bacteria were down-regulated after the gene knocked out. | L399 |
| L367:Replace “decreased by 3.4% compared with” by “were 3.4% lower than”. | Thanks for your suggestion, we have revised it in the text. | L398 |
| L371: “the depletion of these two immune genes in BSFL reduced AMP and ROS production, leading to the decrease in symbiont bacteria and increase in pathogenic bacteria”: It is clear that the reduced expression of these genes reduced AMP and ROS and in this way resulted in an increase in pathogenic bacteria. However, it is not clear to me that the reduction of the symbionts is a direct result of the reduced gene expression, as you seem to indicate. Could it be the case that the gene expression was reduced, that therefore that pathogens were not reduced and that as a consequence of that latter fact the symbionts could not dominate due to competitive exclusion by the outnumbering pathogens? In other words, the reduced expression leads to an increase in pathogens and this in its turn leads to a reduction in symbionts, and not (as you write) a reduced expression leading to a reduction in symbionts and this in its turn to an increase in pathogens. Please clarify very well what is cause and what is result. | Thanks for your suggestion.As we see in figure7, pathogenic bacterial account for only a small fraction of the intestinal bacteria of the black soldier flies, But symbiont bacteria make up a large proportion intestinal bacteria of the black soldier flies. A increase in pathogens could not leads to a reduction in symbionts. My logic was that reduced gene expression lead to reduction of the symbionts and increase of  pathogens . | L402-L404 |
| L382: replace “were actively” by “were active”. | Thanks for your suggestion, we have revised it in the text. | L415 |
| L409:“At 4 days, Duox-TLR3 RNAi larvae with a decrease of 12% and 14.8%abundance of Providencia and Dysgonomonasthan control, respectively.” Where is the verb in this sentence? | Thanks for your suggestion,we have revised it in the text. | L445 |
| L413:what do you mean with “minor bacterial”? Please rephrase in proper English. | Thanks for your suggestion,we have revised it in the text. | L452-453 |
| L418:Replace “a minor bacterium” by “ a bacterium with low relative abundance” | Thanks for your suggestion,we have revised it in the text. | L453 |
| L432:Replace “microbiata” by “microbiota”. | Thanks for your suggestion,we have revised it in the text. | L59 |
| L466: Replace “cell” by “cells”. | Thanks for your suggestion,we have revised it in the text. | L496 |
| L473: “By contrast, Duox inactivation leads to uncontrolled ??? of bacteria in the gut”: it seems a word is missing here. | Thanks for your suggestion,we have revised it in the text. | L502 |
| L484: Replace “domain” by “domains”. | Thanks for your suggestion,we have revised it in the text. | L507 |
| L506:“B.Dorsalis” by “B. dorsalis” | Thanks for your suggestion,we have revised it in the text. | L518 |
| L516:“BSF specifically belongs to the γ-proteobacteria”. This is confusing since BSF is not a bacterium. Please rephrase in a correct way. | Thanks for your suggestion,we have revised it in the text. | L527 |
| L519: “Enterobacteriaceae may plays”. This sentence construction is not correct. Please reformulate. | Thanks for your suggestion,we have revised it in the text. | L529 |
| L522:“Providencia spp. are gram-negative bacteria that was one of Enterobacteriaceae family”. Sentence construction is not correct. Please reformulate. | Thanks for your suggestion,we have revised it in the text. | L531 |
| L525:“which could resistant to”. Same remark. | Thanks for your suggestion,we have revised it in the text. | L533 |
| L525: “Meanwhile …” This sentence is too long and difficult to read because it only ends at line 530. Please reformulate. | Thanks for your suggestion,we have revised it in the text. | L534 |
| L536: Replace “P. Aeruginosa” by “P. aeruginosa”. | Thanks for your suggestion,we have revised it in the text. | L560 |
| L541:By contrast, oriental BSF did not exhibit pathology associated with Pseudomonadaceae.” Where do you have this information from? Please refer to literature to support this claim. | The sequencing results showed that the levels of *pseudomonas* increased after the interference, and at the same time increased *Pseudomonadaceae* bacteriacause toxicity to the host, so I assumed that the uninterferenced black soldier flies didn't show any pathology associated with *Pseudomonadaceae*.I did not find some literature to support this claim |  |
|  | | |
| Reviewer #2 | **Author' Response** | **Revised Text** |
| a. The presentation of results is quite confused and it is difficult to follow the flow of the experiments. A language revision is necessary and the paper is full of typos. | Thanks for your suggestion，the results was reformulate and follow the flow of the experiments.We invite foreign Experts Jeffery K. Tomberlin to revise language problem.we corrected all the typos. |  |
| b:The authors seem not to be aware of the current literature. | Thanks for your suggestion,we provide several recent articles in the paper. |  |
| c No information on the criteria for the choice of the bacterial concentrations administered to the larvae is reported. | I refer to Zhi Chao yao's （zhichao yao.,2016）article, he used the concentration of bacteria is 10^8^ CFU/mL, too low concentration of bacteria can not activate the immune system. |  |
| d It is not clear why they used third instar larvae since this is a stage where Duox gene is weakly expressed. | Beause one instar and two instar larvae are too small to have much intestine, fourth-instar  larvae has feathered，The fifth- instar was too old to take out the intestines.that is the reason we chose the third instar larvae. |  |
| e I’m concerned about the interpretation of some experiments. In fact the authors consider only some of the results to draw their conclusions, while others are completely overlooked. Some examples: - Figure 7B: 4 and 8 days have an opposite trend - Some data are discussed although not statistically significant - *Salmonella* spp. and *S. aureus* differently induce the expression of Duox gene | Thanks for your suggestion,we have revised it in the text. |  |
| f:The efficacy of RNAi in insects is strongly debated (see Scott et al., 2011; Terenius et al., 2011). This approach for gene silencing in Hermetia is new and there are no previous studies on this topic. Since most of the experiments of the current study are based on RNAi, further evidence is necessary to support qPCR data and demonstrate the efficacy of RNAi in this insect model. | Thanks for your suggestion, RNAi worked  well in fruit flies，and our lab was able to use crispercas technology to interfere with the larvae of black soldier flies. At present, only QPCR can measure gene expression to prove RNAi interference. I couldn't do the experiment again because I left my work unit. |  |
| g:Duox and TLR3 genes have been cloned starting from a comparison with Drosophila homologs, but Discussion on these two proteins considers *B. dorsalis*. | Thanks for your suggestion, In the discussion section, the comparison is made between the black soldier flies and *B. dorsalis*'s gut bacteria and had nothing to do with the two cloned proteins. | L518 |
| h:The authors should better argue why they focused attention on TLR3. | Thanks for your suggestion,we have revised it in the text. | L66 |
| i:Discussion needs to be focused. In addition, some aspects are not relevant to the current study (see structural characterization of Duox and TLR). | Thanks for your suggestion,we have revised it in the text. |  |
| I:Supplementary information needs to be revised. For example Figure S8 is blurry and it is not possible to read the text. | Thanks for your suggestion, we have revised FigureS8 in the text. |  |
| Review 3# |  |  |
| L54: insects rely…to defend against… | Thanks for your suggestion,we have revised it in the text. | L53 |
| L77:define Bd | Bd stands for *Bactrocera dorsalis* | L78 |
| L80:How does Kumer et al (as a reference) increase ROS? This sentence does not make sense. | Thanks for your suggestion,we have revised it in the text. | L83-L85 |
| L85： What is expression through an? Again, poorly constructed sentence. | Thanks for your suggestion,we have revised it in the text. | L88 |
| L105：its relationship… | Thanks for your suggestion,we have revised it in the text. | L113 |
| L121 ：replicated three times. | Thanks for your suggestion,we have revised it in the text. | L132 |
| L123：A period is required “….detected.” | Thanks for your suggestion,we have revised it in the text. | L134 |
| L143:distilled water that was then stirred… | Thanks for your suggestion,we have revised it in the text. | L155 |
| L143:What is meant by “and used overnight”? | Thanks for your suggestion,we have revised it in the text. | L156 |
| L135:What is meant by “Pathogen counts were determined using a selective enrichment.”? Were the samples enriched first? If so, how were counts made? | I made a mistake in spelling. We have revised it in the text. | L135 |
| L154:The mean Salmonella spp (CFU/g) of triplicate plates was determined. | Thanks for your suggestion,we have revised it in the text. | L157 |
| L159:… by using TRIzol… according to manufacturer instructions. | Thanks for your suggestion,we have revised it in the text | L166 |
| L160:a full listing of the supply company used is required. | Thanks for your suggestion,we have add full company name in the text. | L165 |

| L164and194:a full and accurate listing of the supplies and company used is required. | Thanks for your suggestion,we have add full company name in the text. | L165,L171, L206 |
| --- | --- | --- |
| L195:amplification program consisted of… | Thanks for your suggestion,we have revised it in the text. | L208 |
| L205:there are multiple primers listed in this reference please be specific which primers were used. | Thanks for your suggestion,we have revised it in the text. | L218 |
| L290:what is meant by “in the natural environment” and “under normal pig manure conversion circumstances” this was not adequately explained in the methods. | L117, Thanks for your suggestion. we have revised it in the text.  “the natural environment” and “normal pig manure conversion circumstances”  means zoonotic pathogenic bacteria *S. aureus* and *Salmonella* spp are naturally  exist in pig manure , not be added artficially. | L305 |
| L306:The graphs do support a complete loss of inhibitory effect until after 2 days. They do support a reduction in the inhibitory effect seen with Fig 1. | Thanks for your suggestion,we have revised it in the text. | L324-L326 |
| L324:Be careful of your wording and punctuation, it appears you are saying the both Salmonella and Staph induced 4.05-times the Duos gene at 12 hours and this is not reflected in the graph. | Thanks for your suggestion,we have revised it in the text. | L345-L348 |
| L325: The 24 hr increase is not shown on the graph? Put the significance designations directly above the error bars not at the top of the graph? Not sure students T-test is the proper analyses nor presentation by histogram. | The 24 h gene expression was shown on the bar graph, and the 24h gene expression returned to normal.Thanks for your suggestion,we have revised Figure3. We compare all the sample groups using the ANOVA analysis. | L216 |
| L326:I do not see on the graphs where Salmonella shows significant difference in Duox? Are you talking about 4 and 8 hours, if so where are the significance designations for Salmonella? Please clarify your statement. | *Salmonella spp* did not activate the *Duox* gene at any point  significantly.Thanks for your suggestion,we have revised it in the text. | L347 |
| L340:Analyses for quantifying effector gene dif is not described in the Methods section; nor is cecropin, ubiquitin and stomoxyn. | Thanks for your suggestion,we have revised it in the text. | L201-203 |
| L349:An example that egfp RNAi and egfpRNAi is presented in these two different ways, with and without spacing. Be consistent throughout the manuscript. Also correct any capitalization of the species name (such as in Ln # 506, 536). | Thanks for your suggestion,we have revised it in the text. | L383 |
| Figures 8 and 9 – the labels and legends are too small, and the resolution of the figures is too low. These require better quality graphics. Dendograms should be shown to demonstrate the relatedness of the community composition. | Thanks for your suggestion, we revised figure 8.figure 9 was pretty clear .It's the upload reason that's making it blurry. |  |
| L409:incomplete sentence. | Thanks for your suggestion,we have revised it in the text. | L445 |
| L420：Where did you demonstrate that the relative abundance of Comamonas was directly tied to reduced ROS and AMP? | On the fourth and eighth days, the Comamonas content in the ds*Duox-TLR3* RNAi group was higher than that of the *egfp*RNAi group, which maybe was due to the decreased level of ROS and AMP. | L455 |
| Fig 8 – Again keep axis values the same so direct comparisons can be made. Put significance markers on these graphs. | Thanks for your suggestion,we have revised Figure 8. |  |
| L429:Tie these last three sentences in better to the immune expression theme and the observations in your study. | Thanks for your suggestion,we have revised it in the text. | L57 |
| L461:This is true only at Day 8 and with Chao and ACE. So this blanket statement is incorrect. | Thanks for your suggestion,we have revised it in the text. | L491 |
| L471:fix spacing | Thanks for your suggestion,we have revised it in the text. | L505 |
| L473: What does uncontrolled of bacteria in the gut… mean? | Thanks for your suggestion,we have revised it in the text. | L502 |
| L516:…belonging… | Thanks for your suggestion,we have revised it in the text. | L526 |
| L518:…could potentially… | Thanks for your suggestion,we have revised it in the text. | L520 |
| L520:play | Thanks for your suggestion,we have revised it in the text. | L529 |
| L522, 524, 525-528, 562 poor sentence structure | Thanks for your suggestion,we have revised it in the text. | L531-L535 |
| L531:need a reference | Thanks for your suggestion, we provide a reference in the text. | L558 |
| L389, 424, 438, 446, 498, 549avoid the first person “We” | Thanks for your suggestion, we have revised it in the text. | L476，468,473 ,428 |
| Reviewer #4: |  |  |
| The following statement from the abstract is misleading: “Currently, it is the only insect approved for such purposes in Europe, Canada, and the USA”. A number of other species of insects can be legally used for feeding animals. | Thanks for your suggestion, we have revised it in the text. | L20 |
| L274: The R packages used should be cited in accordance with R software citation practices. These proper citation can be found in the software library. | Thanks for your suggestion, we have revised it in the text. | L289 |
| L316-319: why would these genes be less expressed at specific intermediary instars? What could be the explanation for that? | Thanks for your suggestion. Our results showed the two genes expressed less at specific intermediary instars, but we do not know why. We speculated that the two genes have different expression level to meet their different functions at different stages of black soldier fly development.We have revised it in the text. | L336-L341 |
| L328-331: These results show that gene expression is increased. When the genes do express you refer to a plot where you show the increase in H_2_O_2_, but don’t discuss this in the text. | *Staphylococcus aureus* activates the *Duox* expression at 12 hours, which is consistent with H_2_O_2_ gene expression being up regulated at 12 hours, as I described in text. | L344-346 |
| Could the authors explain more clearly the mechanism by which the gene expression reduces the microbial load? Is it only H_2_O_2_, or was that the only measurable bioactive compound? | Thank you for your good questions.  About the mechanism by which the gene expression reduces the microbial load. Using RNA interference methods We find gene *BsfDuox and TLR3* could regulate BSFL gut bacteria load. Through analysis gut bacteria density, community composition between RNAi and normal larvae we find *BsfDuox and TLR3* could regulate the homeostasis of the gut bacterial, increase symbiotic bacteria, such as *[Dysgonomonas](F:/MbPL201805212/MbPL201805212/Treat3/B07_taxa_summary/taxa_summary_plots/javascript:gg('g__Dysgonomonas');)* and *Providencia,*  control the bacterial load and decrease the pathogens load in the midgut. Our experiments showed *BsfDuox and TLR3* could inhibit the pathogens and keep gut microbe homeostasis through expressing H_2_O_2_ and AMP. We found *BsfDuox* and *TLR3 are important genes and* H_2_O_2_ and AMP are important bioactive componds in regulating gut microbe homeostasis and inhibiting zoonotic pathogens. But there may be other immune gens and bioactive compounds help to regulate gut microbe homeostasis and inhibiting zoonotic pathogens. That will be our next work. We explained the mechanism in Disccusion Section. | L372 |
| Is there any insight into how activation of these genes reduces activation of other genes as a tradeoff, that might reduce growth performance in farming? In other words, what is the burden on the animal? Is this later described in line 438? | Whether activation of the *Duox* and *TLR3* genes reduces the expression of other genes is not known.The Line 438(Now 468) of text are just extrapolations from the sequencing results.  . | L468 |
| L349-375: show what as observed regarding time to recover expression of certain genes. Could the authors explain why the larvae needs the length of time observed to recover from silencing? What activities are underway that take the observed number of days to recover normal activity? Maybe this is explained in Lines 376-386, but it is not written in a way that is clear to me if this connection is being made. | Thank you for your good questions.  After genes were interferenced we need suitable time to see the role of the genes. The RNAi results showed that the genes silence time is from day 4^th^ to day 8^th^ . The silence time is consistent with the pathogen inhibited time of normal black soldier fly larvae. During 4-8 days we could easily see the role of *Duox* and *TLR3* genes on gut microbes, ROS, AMPs expression and pathogens. Yes We give more explaination in Lines 376-386(Now is Line 405-417). | L405-L417 |
| L432: a comparison is made to mosquitos. I find these types of comparisons relevant since they show how BSF act compared to other species. More clear comparisons with other species and how they react, especially quantitative ones, would be welcome. In particular, it would be very valuable to see how much H_2_O_2_ BSF produce relative to other members of Diptera under similar conditions. Do we see much higher output, which corresponds to their well-known robustness at the larval stage? | Thank you for your positive comment for our results comparisons with insect species. For example Xiao report *Duox* in *Aedes aegypti* and *Drosophila melanogaster* produced ROS( H_2_O_2_) and regulate microbes homeostasis in the their gut.  They quantitatively assay the H_2_O_2._ But their assay methods are different with us. We could know which one expressed H_2_O_2_ is higher. We speculate that the black soldier flies produce more H_2_O_2_ than other diptera insects, which corresponds to their well-known robustness ability to inhibit pathogens at the larval stage in rich pathogens manure environments compare to *Aedes aegypti* and *Drosophila melanogaster*. |  |
| Much is made of BSF’s ability to manage pathogens in industry. Could you please use part of the discussion to give direction to where more work needs to be done to confirm if the ability of BSF to reduce pathogens can be relied upon to produce safe raw larvae for the feed and food industries? Or can you explain how your results show that BSF will not be able to completely resolve a serious safety issue and that other steps will be needed, like heat treatment. | Thank you for your very good questions. Our objective is to use BSFL to convert organic waste (such as livestock and poultry manure) into BSFL protein and biofertilizer. During conversion BSFL could inhibit the most of zoonotic pathogens in BSFL and residues. Moreover, we further use BSFL as feed and food materials we should dry BSFL by heat and grind it into powder. So we could produce safer raw larvae for the feed and food industries. |  |
