# Supplementary material for: Dual oxidase Duox and Toll-like receptor 3 TLR3 in the Toll pathway suppress zoonotic pathogens through regulating the intestinal bacterial community homeostasis in Hermetia illucens L
Source: PLoS One. 2020 Apr 30;15(4):e0225873. doi: 10.1371/journal.pone.0225873 (PMC7192390; doi:10.1371/journal.pone.0225873)
Supplement: Attachment — Submitted filename: Response to Reviewers.docx [file pone.0225873.s010.docx]

Dear Editor,

Journal of PLoS One

**Subject: Response to Reviewers’ Comments**

Kindly refer to your email sent on 2020/02/06 regarding the reviewers’ comments on our manuscript ID WM-19-31548 entitled "Dual oxidase Duox and Toll-like receptor 3 TLR3 in the Toll pathway suppress zoonotic pathogens through regulating the intestinal bacterial community homeostasis in *Hermetia illucens* L.". Based on his/her kind comments, we have carefully modified the manuscript (The revised text is marked with tracked change) and response to reviewers’ comments carefully. Thank you very much for the encouragement and providing very useful suggestions to improve the quality of our manuscript. We hope that the revised-manuscript now will meet the standards for publication in your esteemed journal.

Thank you very much.

Sincerely yours,

**Corresponding author**

Dr. Zhang Jibin

Professor

College of Life Sciences and Technology,

Huazhong Agricultural University,

Wuhan 430070, P.R. China

E-mail: [zhangjb@mail.hzau.edu.cn](mailto:zhangjb@mail.hzau.edu.cn)

Reviewer #2: This study focused on the mechanism black soldier fly larvae used to inhibit two pathogens, S. aureus and Salmonella. To do so, two immune genes were silenced, upon which two symbionts were observed to decrease in abundance and inhibiting effects on the pathogens were reduced. This demonstrated that the immune genes under study play a role in the pathogen reducing effect of native BSFL.
The paper brings important and new information and is highly relevant for the application of BSFL as waste converter, since waste streams can contain the pathogens under study. However, the language use was very poor and disappointing, and it reduced the readability of the paper. The many language errors deduct the reader from the content and devaluate the manuscript. As a reviewer, I corrected a number of language errors, but certainly not all of them. I strongly recommend professional language editing. On some places in the paper, statements were formulated too general and conclusions were drawn too strong in my opinion, i.e. based on evidence that was not substantial enough to make that conclusion. Also some elements are missing in the materials and methods section. I believe this manuscript can become a very meaningful paper as it contains interesting results but it needs major revisions.

**Response to reviewer 2 comments**

a. Bacterial concentration. Yao Z. et al., 2016 is a study on B. dorsalis, a different insect species. Why did the authors refer to this paper for their experiments on BSF? Did they perform preliminary assays in BSF to demonstrate that “low concentrations of bacteria cannot activate the immune system”, as stated in the rebuttal letter?

**Response:** Thank your good question. Because *Hermetia illucens* and *B. dorsalis* is close relatives and both belong to Diptera. Their *BsfDuox* and *BsfTLR3* were similar. They did not perform preliminary assays in BSF to demonstrate that “low concentrations of bacteria cannot activate the immune system”. We just did some preliminary experiments to explore the suitable concentrations of bacteria for BSFL infect treatment.

b. I still do not understand why third instar larvae were used (see answers as “four instar larvae were feathered”??? “fifth instar larvae were too old to take out the intestine”???)

**Response:** The third instar is the right size to pull out the intestines. The other larvae are either too small or have no intestines to pull out.

c. Interpretation of some experiments. I do not see any information in the new text about the points that I indicated in the previous revision (see trend of some markers, statistics, expression of Duox gene, etc.

**Response:** Thanks for your suggestion, we have revised it in text(L130-133, L154-L155, L164-171, L183-L184, L190-L193, L199-L203, L212-L213, L224, L228-L229, L233-L236, L239-240, L245-L246, L247-L254, L270-L271, L283, L327-L328).

d. RNAi. The authors just say that RNAi works in Drosophila, they declare to be able to use Crispr/cas9 gene editing in Hermetia and that they could not do any additional experiments since one of them left the laboratory. What does this mean? This does not reply to my question on RNAi. This is the first report on the use of RNAi in BSF, thus I think that this aspect is worthy of a deeper investigation.

**Response:** Our experiment is to study the black soldier flies, using crisper-cas system and RNAi technology to interfere the genes of BSF have achieved good results(Zhan et al 2019). RNAi technology worked well in *Drosophila* and *Caenorhabditis elegans*. *Schistocerca americana* et al. RNAi also has drawbacks and obscure points, However, such as those related to differences in species sensitivity,there are hundreds of research papers to prove the effectiveness of RNAi in insects. The QPCR test could verify the RNAi results, each QPCR experiment was repeated three times.

e:Some of the revised points indicated in the cover letter are not present in the text (see for example L518 for B. dorsalis or L66 for TLR3)

**Response:** Thank you for your suggestion. In L518 I wrote Enterobacteriaceae bacteria instead of a B. dorsalis ,which appears in the cover letter. In L66 I wrote *TLR3*,which also appears in the cover letter.

f:Although Discussion has been revised, it still contains some information that, in my opinion, it not relevant to the current study (see for example structural characterization of Duox and TLR). Moreover, I’m still convinced that the whole section should be more focused.

**Reponse:** Thank you for your suggestion. We have deleted not relevant contents in discussion section such as structural characterization of Duox and TLR and other not relevant information(L549，L599-L603，L621-L627，L643).

g: Many typos are still present in the text.

**Reponse:** Thanks for your suggestion, We have double checked and revise it in whole text(L535，L539 ,L663-L888 et al.).
